# Reducing underreporting of abortion in surveys: Results from two test applications of the list experiment method in Malawi and Senegal

**DOI:** 10.1371/journal.pone.0247201

**Published:** 2021-03-03

**Authors:** Heidi Moseson, Ruvani Jayaweera, Sarah Huber-Krum, Sarah Garver, Alison Norris, Caitlin Gerdts

**Affiliations:** 1 Ibis Reproductive Health, Oakland, CA, United States of America; 2 Harvard T. H. Chan School of Public Health, Cambridge, MA, United States of America; 3 University of Chicago, Chicago, IL, United States of America; 4 The Ohio State University, Columbus, OH, United States of America; University of Cape Coast, GHANA

## Abstract

**Background:**

Accurately measuring abortion incidence poses many challenges. The list experiment is a method designed to increase the reporting of sensitive or stigmatized behaviors in surveys, but has only recently been applied to the measurement of abortion. To further test the utility of the list experiment for measuring abortion incidence, we conducted list experiments in two countries, over two time periods.

**Materials and methods:**

The list experiment is an indirect method of measuring sensitive experiences that protects respondent confidentiality by hiding individual responses to a binary sensitive item (i.e., abortion) by combining this response with answers to other non-sensitive binary control items. Respondents report the number of list items that apply to them, not which ones. We conducted a list experiment to measure cumulative *lifetime* incidence of abortion in Malawi, and separately to measure cumulative *five-year* incidence of abortion in Senegal, among cisgender women of reproductive age.

**Results:**

Among 810 eligible respondents in Malawi, list experiment results estimated a cumulative lifetime incidence of abortion of 0.9% (95%CI: 0.0, 7.6). Among 1016 eligible respondents in Senegal, list experiment estimates indicated a cumulative five-year incidence of abortion of 2.8% (95%CI: 0.0, 10.4) which, while lower than anticipated, is seven times the proportion estimated from a direct question on abortion (0.4%).

**Conclusions:**

Two test applications of the list experiment to measure abortion experiences in Malawi and Senegal likely underestimated abortion incidence. Future efforts should include context-specific formative qualitative research for the development and selection of list items, enumerator training, and method delivery to assess if and how these changes can improve method performance.

## Introduction

Accurate epidemiological surveillance of abortion (induced termination of pregnancy) is crucial for developing informed and responsive family planning programs, policies, and interventions [1]. However, abortion is highly stigmatized in many settings, complicating reliable measurement of its occurrence [1–4].

Methodologies commonly used to estimate abortion incidence have widely acknowledged limitations [3,5–8]. Direct measurement methods, whereby respondents are asked directly about their abortion experience(s), may result in underreporting due to social desirability bias and privacy concerns [2,3]. Underreporting is likely magnified in contexts where abortion is legally restricted [3,5,7,9], due to fears for personal safety and desires to avoid legal or social repercussions. Indirect methods for measuring abortion, such as the Abortion Incidence Complications Methods (AICM), the Anonymous Third Party Reporting (ATPR) method, and the Network Scale-Up Method (NSUM) [10–12], rely on sources of information other than the individual who had the abortion, and until recently, the AICM may have excluded abortions that took place outside of the formal health-care system, such as self-managed abortions [6,13]. Further, these methods require large and costly data collection efforts [10–12]. Despite these limitations, these indirect methods in some cases could be the only available sources for country-level estimates in settings where abortion is restricted.

In response to these limitations, researchers have recently applied an indirect method known as the list experiment to estimate abortion incidence. The list experiment method asks a respondent to identify *how many* items on a list of health events the individual has personally experienced. Respondents do not disclose *which* events they have experienced, only how many. Respondents are randomized to respond to either a list of non-sensitive control items only or the same control list plus the sensitive item under study (i.e., abortion). By relying directly on individuals’ reports (as opposed to clinicians or confidantes), the list experiment may be better positioned than other indirect methods to capture all abortion experiences, including those that the respondent has not disclosed to confidantes, and that take place in a clinic setting or are self-managed.

Beginning in 2015, the list experiment method has begun to be used to measure abortion incidence [14–17], with mixed results. List experiments to measure abortion in Iran, Liberia, Pakistan, the United States, and Texas [14,16–20], showed promising results for reducing underreporting, while list experiments from India, Tanzania, Turkey and Vietnam have failed or shown inconclusive results [21–24]. Thus, additional empirical data on the performance of the abortion list experiment in various contexts, including its strengths and limitations, are needed.

To further test the utility of the list experiment for measuring abortion incidence over various time frames and in new contexts, we applied the methodology in two countries where abortion is legally restricted and abortion data are limited: Malawi and Senegal. In both countries, abortion is prohibited by law except to save the pregnant person’s life; in practice, it is rare for anyone to meet the legal requirements for abortion [25,26]. Recent abortion estimates in each country are from indirect methods–in Malawi, the estimated abortion rate is 38 abortions per 1,000 reproductive aged women per year (2015) [27]; in Senegal, the estimated abortion rate is 17 abortions per 1,000 reproductive aged women per year (2012) [28]. These likely underreport the true incidence of abortion in each country, as the indirect method utilized to generate these estimates relied on third party recall and assumptions about care-seeking from healthcare professionals [28]. Given lower than expected abortion incidence estimates in each country, we set out to test the performance of a newer, indirect method of abortion measurement–the list experiment–to explore its ability to reduce underreporting of abortion in these restrictive contexts.

## Materials and methods

### Ethical approval

The study in Malawi was approved by the institutional review boards at Ohio State University IRB and the Malawi College of Medicine. The study in Senegal was reviewed and approved by the Comité National d’Ethique pour la Recherche en Santé based in Dakar, Senegal. Participants provided verbal informed consent.

### Study participants

In Malawi, we utilized data from the third wave of Umoyo wa Thanzi (UTHA), a community-based two-stage stratified cluster cohort study of reproductive-aged cisgender women (ages 15–49) in rural Lilongwe, Malawi (conducted October 2016–April 2017). In Senegal, we utilized data from a stratified, probability proportional to size sampled household survey of cisgender women (ages 15–44) in four regions of the country: Dakar, Diourbel, Louga and Ziguinchor (conducted in September-October 2017). More details of sampling for both surveys are reported elsewhere [29].

As the primary aims of both studies were to provide descriptive statistics in relation to family planning outcomes, sample size was not estimated to achieve a stated power for any given statistical test. Toward the goal of estimating abortion incidence with some precision, a sample size of 1000 individuals was estimated necessary to reach approximately 85 individuals with a history of induced abortion in the past five years (based on the lowest abortion incidence estimate from both sites). This sample size would allow for a population-level estimate of induced abortion with a margin of error of 10% [28].

### Survey design and administration

Trained enumerators conducted survey interviews face-to-face in Chichewa in Malawi, and in French and Wolof in Senegal (cisgender men and cisgender women enumerators in Malawi; cisgender women enumerators only in Senegal). Survey data were entered electronically on tablets.

**Fig 1:**
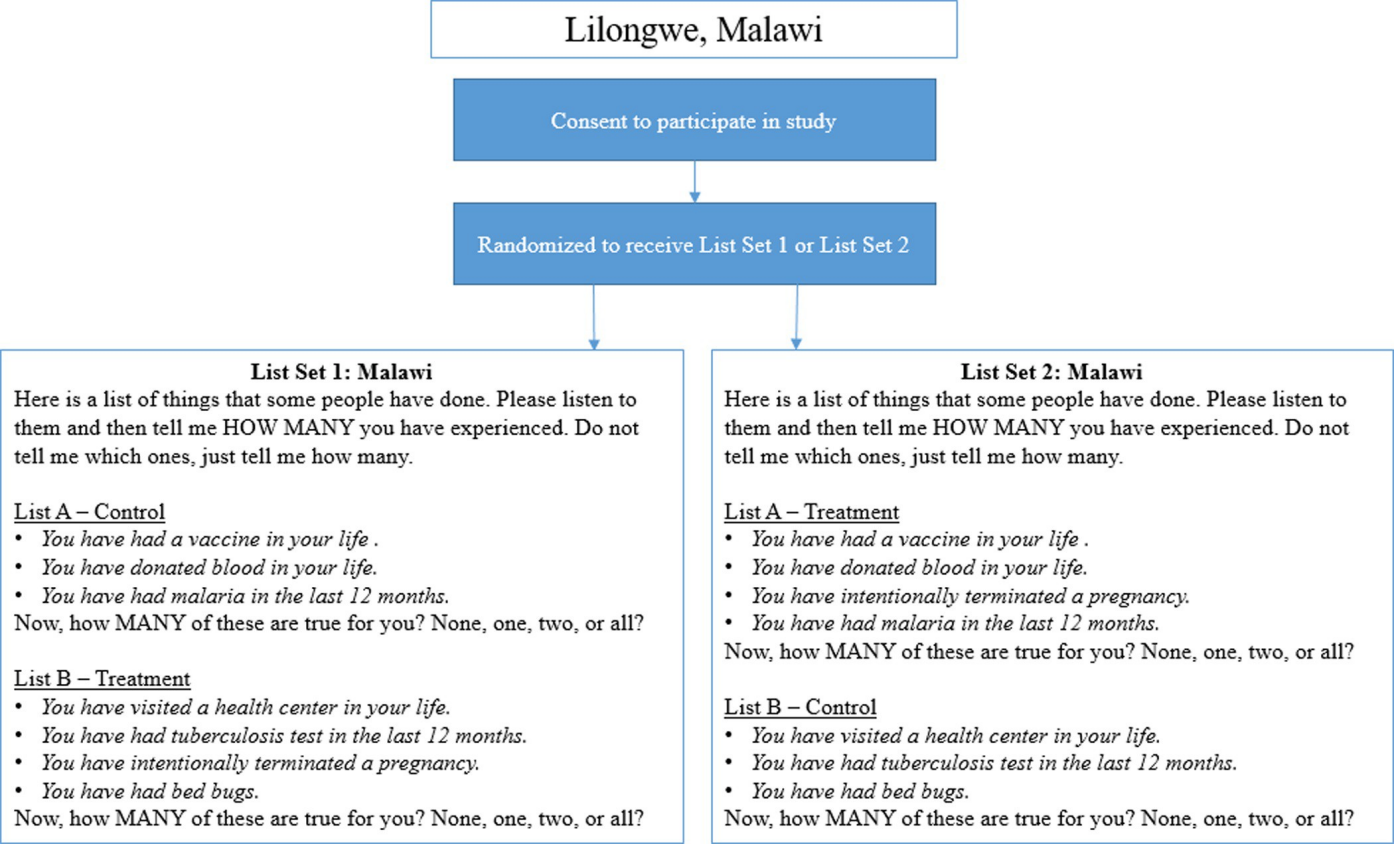
Diagram indicating participant randomization to one of two list experiment sets, as well as the full text of the list experiment questions. List experiment sets presented below for the Lilongwe, Malawi survey.

**Fig 2:**
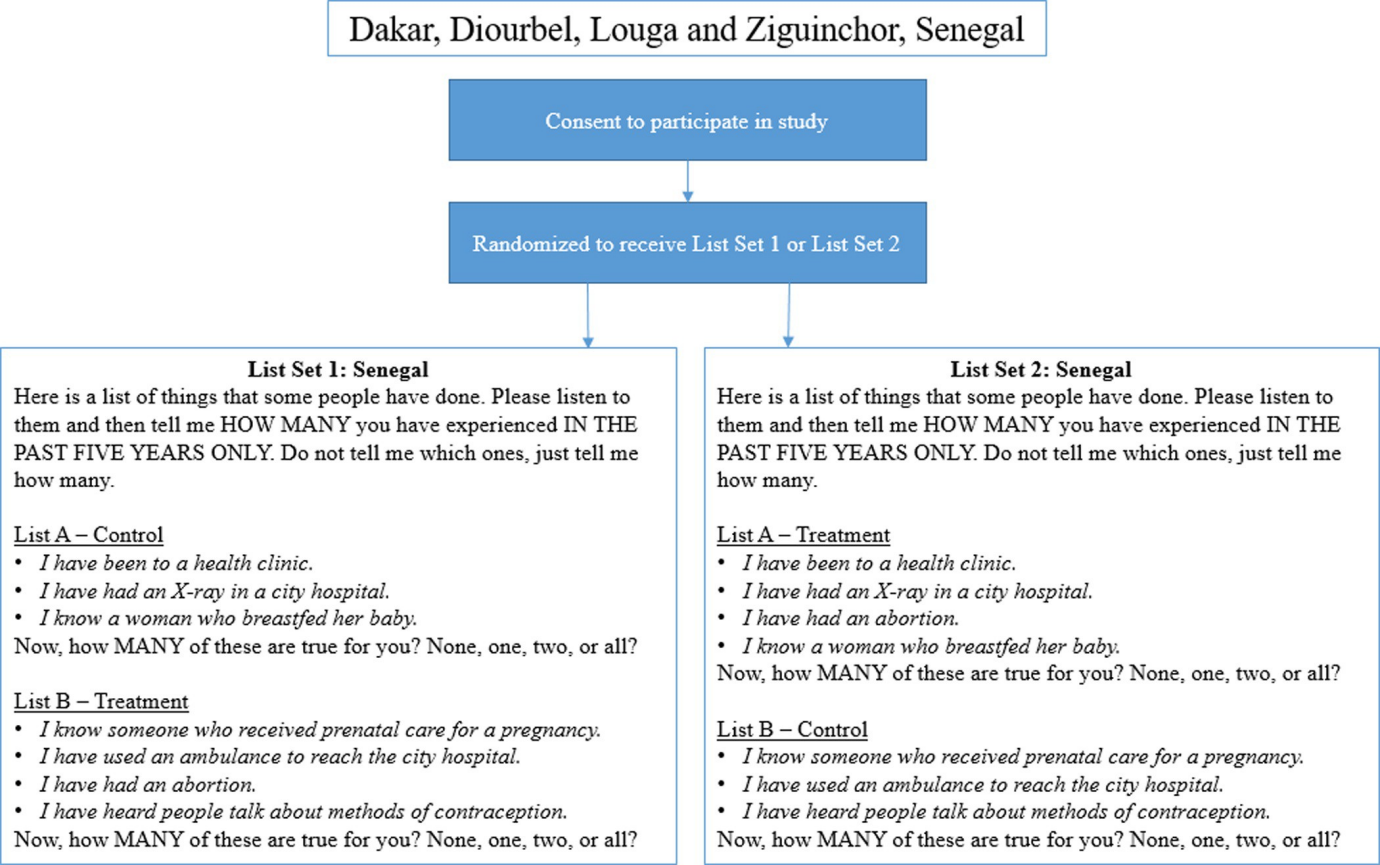
Diagram indicating participant randomization to one of two list experiment sets, as well as the full text of the list experiment questions. List experiment sets presented below for the Senegal survey.

Survey instruments included questions on socio-demographic characteristics, reproductive history (including abortion), list experiment questions (Figs 1 and 2), and contraceptive use.

For the primary outcome, to measure the proportion of the population that had ever had an abortion (Malawi), or that had had an abortion in the past five years (Senegal): we utilized a double list experiment, a variant of the standard list experiment in which two lists are used, rather than one. For the two control lists, the research team selected control items utilizing country-specific Demographic and Health Survey (DHS) data. To minimize variance, control items were selected to be (1) negatively correlated *within* a list (to avoid ceiling or floor effects, whereby a respondent has experienced either all or none of the events on a list, respectively, as this would invalidate confidentiality); and (2) positively correlated *across* lists, so a respondent who has experienced a control item on one list is likely to have experienced a control item on the second list [30].

The sensitive item, abortion, was randomized on a 1:1 basis to appear on either the first or second control list. In this way, each participant received both lists: half of the sample received the first list with control items only, and the second list with control items plus abortion, while the other half of the sample received the first list with abortion added, and the second list with control items only (Figs 1 and 2).

At the time of survey administration, enumerators first administered a practice list to each respondent that asked about foods eaten in the past week to ensure respondent comprehension of the list experiment method. After insuring participant comprehension of the format, the enumerator then read the two main lists separately to the respondent, and asked the respondent to report the *number* of experiences that applied to them for each list [30].

### Analysis

For both countries, we estimated the cumulative incidence of abortion over the specified time period (lifetime or past 5 years). For both list sets, we calculated the average number of items reported for the control and treatment version of each list. Next, we subtracted the mean number of items reported for the control list from the mean number of items reported for the treatment list, separately for List A and List B. We then averaged these two difference-in-means calculations to provide a more precise estimate of the population proportion that has had an abortion [14,30].

Two identifying assumptions underlie this estimator: 1) the inclusion of the sensitive item does not affect respondents’ answers to the control items (no design effect), and 2) respondents answer the sensitive item truthfully (no liars) [31]. To evaluate these assumptions, we tested for a design effect. A design effect exists if the number of control items that an individual reports depends on whether or not the list includes the sensitive item (abortion)–i.e., if participants were so concerned about the presence of abortion on the list, they would falsely report a lower number of *control* item experiences to make especially clear that they have not had an abortion. To assess for a design effect, we calculated the difference between the lists with and without abortion in the proportions of participants with at least *X* positive responses [30]. Negative differences suggest the potential presence of a design effect [31]. We then conducted a likelihood ratio test using the R ‘list’ package to formally identify or reject the presence of a design effect [32]. Sensitivity analyses explored whether list estimates varied by enumerator gender. Data management and analyses were conducted in Stata 15 and R, and estimates were weighted based on sampling design.

## Results

### Study participants

We included 810 respondents in analyses for Malawi, and 1016 respondents in Senegal (Table 1). Per the double list experiment format, each of these respondents received one list with the sensitive item (abortion) included, and one list without the sensitive item. We excluded any respondents outside of the eligible age range, or who did not provide responses to both treatment and control lists (n = 53 in Malawi, n = 0 in Senegal). Consistent with general population characteristics in both sites, most participants were between the ages of 21 and 35 years, and over half had attended primary school or less.

**Table 1:** Selected sociodemographic characteristics of a probability sample of participants in a household survey of cisgender women of reproductive age in Lilongwe, Malawi (n = 810), and Dakar, Diourbel, Louga and Ziguinchor regions in Senegal (n = 1,016).

	Malawi (N = 810)			Senegal (N = 1,016)		
		%	%		%	%
	N	Unweighted	Weighted	N	Unweighted	Weighted
**Age (years)**						
Less than 18	9	1	1			
18–20	65	8	8	71	7	6
21–25	218	28	28	236	23	23
26–30	155	20	20	196	19	20
31–35	177	23	23	222	22	21
36–44	152	20	20	291	29	30
Greater than 44	3	0.4	0.4	--	--	--
Missing	31	--	--	0	--	--
**Education**						
None	59	7	7	327	32	21
Primary	530	66	66	302	30	34
Secondary	213	27	27	290	29	30
Post-secondary	--	--	--	97	10	15
Missing	8	--	--	0	--	--
**Relationship status**						
Single, never married	31	4	4	188	19	24
Unmarried, living with partner	--	--	--	6	1	1
Married, living with partner	625	76	76	544	54	52
Married, not living with partner	48	6	6	216	21	15
Divorced/separated	88	11	11	50	5	6
Widowed	13	2	2	12	1	2
Missing	5	--	--	0	--	--
**Religion**						
Muslim	5	0.7	0.7	982	97	96
Christian	612	88	88	34	3	4
Other	81	12	12	0	0	0
Missing	112	--	--	0	--	--

### List experiment estimates

List experiment results from Malawi yielded an estimate of the lifetime cumulative incidence of abortion of 0.9% (95% CI: 0.0%, 7.6%; Table 2). In Senegal, list experiment results estimated a cumulative five-year incidence of abortion of 2.8% (95% CI: 0.0%-10.4%; Table 2).

**Table 2:** List experiment estimates of the percentage of cisgender women of reproductive age who have had an abortion in their lifetime (Lilongwe, Malawi) and in the past five years (Senegal).

	List A estimate	List B estimate	Average of Lists A & B	95% CI
**Malawi (n = 810)**				
Unweighted	0.7%	1.6%	**1.2%**	0–7%
Weighted	-0.3%	2.0%	**0.9%**	0–8%
**Senegal (n = 1016)**				
Unweighted	13.4%	6.1%	**9.7%**	4–15%
Weighted	4.5%	1.0%	**2.8%**	0–10%

Results from list experiment estimators, by each list (A and B), as well as combined; 95% confidence intervals are calculated using linear mixed models that account for clustering.

In response to a direct question about lifetime experience of abortion, 4 (0.4%) respondents in Senegal responded affirmatively. For comparison, the five-year weighted list experiment estimate (2.8%) is seven times the magnitude of the abortion estimate obtained from the direct question. In Malawi, the survey did not ask about abortion directly.

### Sensitivity analyses

Despite a number of negative differences between items reported on treatment and control lists (S1 and S2 Tables), the likelihood ratio test rejected the presence of a design effect for all lists (Malawi: List A: *p* = 0.75, List B: *p* = 0.76; Senegal: List A: *p* = 0.37, List B: *p* = 0.99). However, the difference in ceiling effects (respondents reporting that all list items were true for them) for List A in both Malawi and Senegal may warrant attention. In Malawi, 14% of respondents to the control version of List A reported ALL items being true for them, versus only 0.8% of those who received the treatment version; similarly in Senegal, 16% of respondents to the control version of List A reported all items being true for them, versus only 3% who received the treatment version of List A. The results of the design effect test suggest that we do not have sufficient evidence to rule out chance as a cause for this, but the difference could reflect that some respondents may have altered responses based on the presence of the sensitive item on the list. We found no difference in abortion incidence estimates when stratified by enumerator gender in Malawi (in Senegal, all enumerators were cisgender women).

## Discussion

Using a recently introduced methodology for measuring abortion–the list experiment–we estimated that 0.9% of reproductive age women in Malawi had ever had an abortion, and that 2.8% of reproductive age women in Senegal had an abortion in the past five years. However, given knowledge of family planning and fertility statistics in both countries, these list experiment results are likely underestimates of the true incidence of abortion [27,28]. Indeed, based on prior AICM estimates of induced abortion in Malawi and Senegal and United Nations estimates of the population of women of reproductive age in each country for 2016, we would expect that at least 3.0% of Malawian women per year would have had an abortion, and 1.7% of Senegalese women per year, or 8.5% over five years [27,28,33]. This five-year figure for Senegal is closer to the unweighted list experiment estimate of 9.7% from respondents in Senegal. While the list experiment in estimate in Senegal reduced underreporting of abortion as compared to a direct question, it nonetheless may still have resulted in substantial underreporting. There are a number of possible explanations for these lower than hypothesized weighted estimates, falling into three general categories listed here in order of greatest likelihood: (1) design issues with the list experiment, (2) pervasive sensitivity bias preventing honest disclosure of abortion, and (3) chance.

The most important step in designing a list experiment is selecting appropriate individual list items. Ideally, an investigator would select control list items that are reasonably related to the sensitive item, to minimize the sensitive item “jumping out” from the others, and potentially causing respondents to alter their responses as a result (a design effect) [30]. However, given the social sensitivity around nearly all sexual and reproductive health services in the settings under study, it was difficult to identify related health experiences comparable to abortion that were not themselves also sensitive or stigmatized. This led us to select control items from a broader range of health experiences, including some more widely experienced in the population than ideal [30], which may have altered respondents’ decisions to report experiences truthfully (evidenced by discrepancies in ceiling effects across treatment versus control lists).

It is also plausible that respondents were not familiar with individual list items, and thus could not answer accurately. For instance, the concept of an “x-ray” may have been foreign to some participants. While enumerators encouraged all respondents to ask clarifying questions, it is possible that some did not. Cognitive interviews conducted in the United States found that individual list item comprehension is challenging for list experiments measuring abortion [18]; the same may have been true in Malawi and Senegal. Survey enumerators in Malawi reported that participants often experienced challenges in responding to the list items correctly, with some participants answering “yes” or “no” after each item was read, despite practice questions and repeating the question. It may even be possible that some interpreted the word “abortion” differently. With the increasing availability of abortion medications, coupled with estimates that 21% of abortions in Senegal are self-managed [28], it may be that people do not view self-managed medication abortion as “abortion”. Perhaps, medication abortion is viewed as a form of menstrual regulation or by another different name if it occurs outside of the healthcare system [34]. If this were true, respondents may not have counted these experiences in their tallies.

Compounding these issues, it is possible that respondents had poor comprehension of the unique list experiment format. The question requires respondents to keep a mental tally of health experiences over a specified period. In study samples with low numeracy, this could impact the accuracy of data collected, thereby biasing the estimates. Future surveys could include measures of numeracy to explore if and how list experiment responses vary with numeracy. It may also be possible that participants do not perceive the list format as anonymous; intentional statements from enumerators in question scripts could address this in future surveys.

Beyond possible design issues with the lists themselves, these abortion estimates may be low due to the same sensitivity bias that results in underreporting in direct questions of abortion: people fear stigmatization or legal repercussions for disclosing an abortion experience, and thus do not report it [25,29,35]. A 2018 report found that 38% of the female prison population in Senegal is imprisoned for alleged abortion or infanticide [36]; with such powerful legal repercussions, it is plausible that respondents did not feel comfortable disclosing abortion even via an indirect format. A recent review of list experiment results highlighted fear of legal repercussions as an important driver of underreporting in survey research [37]. While we found no evidence for systematic bias in the number of *non-sensitive* items reported (the design effect test), it could be that respondents accurately represented the number of non-sensitive items experienced but simply did not add abortion to their tally of experiences. Even in less legally punitive settings, it is well established that people have reservations about reporting abortion experiences [1,2]. Thus, it seems reasonable to assume that these same concerns could have contributed to the lower than anticipated list experiment estimates. If this is true, the list experiment failed to prevent underreporting of abortion in these two settings.

Alternatively, although unlikely, it is possible that our assumption that the true incidence of abortion is much higher than these estimates is incorrect, or that it is due to chance alone in the samples selected. Perhaps the stigma and legal repercussions act as a deterrent, and people are not inducing abortions as frequently in Malawi and Senegal as in similar contexts elsewhere. Indeed, data on the occurrence of infanticide in Senegal suggests that many people are not able to obtain abortions [29,38]. However, we do not think this is likely given data on high-levels of treatment for abortion complications [39–41].

It is also possible that, by chance, the particular characteristics of our study samples differ from the national population in ways that could explain part of the discrepancy between the estimates we present and national estimates. For instance, in our Malawi study, the sample primarily consisted of married women with relatively good access to contraceptive services at a local community hospital. Given the predominantly rural nature of the sample, it is possible that unwanted birth was viewed more often as a socially safer choice than clandestine abortion. With a more diverse sample, we might have found a higher estimate of abortion incidence.

Future studies that seek to utilize the list experiment methodology should consider these limitations. Possible solutions for future studies include, first and foremost, a recommendation to conduct formative qualitative research in each context to understand the appropriate way to ask about experiences with abortion in the words that individuals would use to describe it. Further, this qualitative research should elicit input on the proposed non-sensitive list items–specifically, to identify items that do not stand out so clearly as unrelated to sexual and reproductive health, and that model negative within-list correlation, and positive between-list correlation. Additionally, rigorous training of enumerators to ensure they understand the purpose of the list format, with key strategies for how to explain the format to participants, is crucial. Positioning the list questions at the start of a survey, particularly when these questions are embedded within a larger survey, may mitigate question fatigue that can lead to data quality and completeness issues. Finally, researchers may want to consider potentially disclosing to participants the objective of the list experiment and explicitly highlighting the protection of confidentiality that this format affords.

Given the paucity of data on abortion incidence and the well-documented challenges of obtaining unbiased data on this essential reproductive health event, the development of innovative measurement tools and methods is of vital importance. The results presented here provide important data points on the performance of a recently introduced method of measuring abortion: the list experiment. Despite some success in reducing underreporting as compared to a direct question, findings highlight limitations of the method and suggest potential process modifications that could increase the accuracy and utility of the method. After implementing the proposed process changes, further testing of the list experiment method with rigorous comparisons to other indirect measurement tools is warranted to evaluate if and when continued use of the list experiment for measurement of abortion is warranted.

## Supporting information

S1 Table Detailed assessment of weighted response proportions by number of reported items to list experiment questions, by list, among respondents in Malawi (n = 810). The objective of this analysis is to identify evidence for a design effect in either list.
(DOCX)

S2 Table Detailed assessment of weighted response proportions by number of reported items to list experiment questions, by list, among respondents in Senegal (n = 1016). The objective of this analysis is to identify evidence for a design effect in either list.
(DOCX)

## References

[1] Singh S, Remez L, Tartaglione A. Methodologies for estimating abortion incidence and abortion-related morbidity: A review. New York, NY: Guttmacher Institute and the International Union for the Scientific Study of Population; 2010.

[2] Lindberg L, Kost K, Maddow-Zimet I, Desai S, Zolna M. Abortion Reporting in the United States: An Assessment of Three National Fertility Surveys. Demography. 2020;57(3):899–925. 10.1007/s13524-020-00886-4 32458318 PMC7329789

[3] Jagannathan R. Relying on surveys to understand abortion behavior: some cautionary evidence. Am J Public Health. 2001;91(11):1825–31. 10.2105/ajph.91.11.1825 11684611 PMC1446886

[4] Lara D, Strickler J, Olvarrieta CD, Ellertson C. Measuring induced abortion in Mexico: A comparison of four methodologies. Sociological Methods & Research. 2004;32(4):529–58. 10.1177/0049124103262685

[5] Anderson BA, Katus K, Puur A, Silver BD. The validity of survey responses on abortion: evidence from Estonia. Demography. 1994;31(1):115–32. 8005338

[6] Singh S, Juarez F, Prada E, Bankole A. Estimating Abortion Incidence: Assessment of a Widely Used Indirect Method. Population Research and Policy Review. 2019;38(3):429–58. 10.1007/s11113-019-09517-2

[7] Jones EF, Forrest JD. Underreporting of Abortion in Surveys of U.S. Women: 1976 to 1988. Demography. 1992;29(1):113–26. 10.2307/2061366 1547898

[8] Jones EF, Forrest JD. Use of a supplementary survey of abortion patients to correct contraceptive failure rates for underreporting of abortion. New York, NY: Population Division, Department of Internaional Economic and Social Affairs, United Nations; 1991.

[9] London K, Williams L. A comparison of abortion underreporting in an in-person interview and self-administered questionnaire. 1990.

[10] Singh S, Prada E, Juarez F. The Abortion Incidence Complications Method: A Quantitative Technique. In: Singh S, Remez L, Tartaglione A, editors. Methodologies for Estimating Abortion Incidence and Abortion-Related Morbidity: A Review. New York, NY: Guttmacher Institute; International Union for the Scientific Study of Population; 2010. p. 71–85.

[11] Rossier C. The anonymous third party reporting method. In: Singh S, Remez L, Tartaglione A, editors. Methodologies for estimating abortion incidence and abortion-related morbidity: a review. New York and Paris: Guttmacher Institute and IUSSP; 2010. p. 99–106.

[12] Sully E, Giorgio M, Anjur-Dietrich S. Estimating abortion incidence using the network scale-up method. Demographic Research. 2020;43:1651–84. 10.4054/DemRes.2020.43.56

[13] Moseson H, Herold S, Filippa S, Barr-Walker J, Baum SE, Gerdts C. Self-managed abortion: A systematic scoping review. Best Practice & Research Clinical Obstetrics & Gynaecology. 2019. 10.1016/j.bpobgyn.2019.08.002 31859163

[14] Moseson H, Massaquoi M, Dehlendorf C, Bawo L, Dahn B, Zolia Y, et al. Reducing under-reporting of stigmatized health events using the List Experiment: results from a randomized, population-based study of abortion in Liberia. Int J Epidemiol. 2015;44(6):1951–8. 10.1093/ije/dyv174 26342584 PMC5156336

[15] Moseson H, Treleaven E, Gerdts C, Diamond-Smith N. The List Experiment for Measuring Abortion: What We Know and What We Need. Stud Fam Plann. 2017;48(4):397–405. 10.1111/sifp.12042 29148056

[16] Moseson H, Gerdts C, Dehlendorf C, Hiatt RA, Vittinghoff E. Multivariable regression analysis of list experiment data on abortion: results from a large, randomly-selected population based study in Liberia. Population Health Metrics. 2017;15(1):40. 10.1186/s12963-017-0157-x 29268794 PMC5740939

[17] Cowan SK, Wu LL, Makela S, England P. Alternative Estimates of Lifetime Prevalence Of Abortion from Indirect Survey Questioning Methods. Perspectives on Sexual and Reproductive Health. 2016;48(4):229–34. 10.1363/48e11216 27513590

[18] Moseson H, Filippa S, Baum SE, Gerdts C, Grossman D. Reducing underreporting of stigmatized pregnancy outcomes: results from a mixed-methods study of self-managed abortion in Texas using the list-experiment method. BMC Womens Health. 2019;19(1):113. 10.1186/s12905-019-0812-4 31481033 PMC6720920

[19] Huber-Krum S, Hackett K, Kaur N, Nausheen S, Soofi S, Canning D, et al. An Application of the List Experiment to Estimate Abortion Prevalence in Karachi, Pakistan. Int Perspect Sex Reprod Health. 2020;46(Suppl 1):13–24. 10.1363/46e0520 33326396

[20] Ghofrani M, Asghari F, Kashanian M, Zeraati H, Fotouhi A. Prevalence of Induced Abortion in Iran: A Comparison of Two Indirect Estimation Techniques. Int Perspect Sex Reprod Health. 2018;44(2):73–9. 10.1363/44e6218 30475213

[21] Bell SO, Bishai D. Can a List Experiment Improve Validity of Abortion Measurement? Stud Fam Plann. 2019;50(1):43–61. 10.1111/sifp.12082 30675727 PMC6619401

[22] Huber-Krum S, Karadon D, Kurutas S, Rohr J, Baykal SS, Okcuoglu BA, et al. Estimating abortion prevalence and understanding perspectives of community leaders and providers: Results from a mixed-method study in Istanbul, Turkey. Womens Health (Lond). 2020;16:1745506520953353. 10.1177/1745506520953353 32853055 PMC7457705

[23] Treleaven E. The list experiment: piloting a methodology to measure stigmatized behaviors around sex-selective abortion in Vietnam. The International Union for the Scientific Study of Population; Oct 29-Nov 3, 2017; Cape Town, South Africa2017.

[24] Elewonibi B, Amour C, Gleason S, Msuya S, Canning D, Shah I. Estimating the lifetime incidence of induced abortion and understanding abortion practices in a Northeastern Tanzania community through a household survey. Contraception. 2020. 10.1016/j.contraception.2020.10.013 33098850

[25] Malawi Penal Code. 1930.

[26] Code Penal Sénégal, Article 305.

[27] Polis CB, Mhango C, Philbin J, Chimwaza W, Chipeta E, Msusa A. Incidence of induced abortion in Malawi, 2015. PLoS One. 2017;12(4):e0173639. 10.1371/journal.pone.0173639 28369114 PMC5378324

[28] Sedgh G, Sylla AH, Philbin J, Keogh S, Ndiaye S. Estimates of the incidence of induced abortion and consequences of unsafe abortion in Senegal. International perspectives on sexual and reproductive health. 2015;41(1):11–9. 10.1363/4101115 25856233 PMC4712915

[29] Moseson H, Ouedraogo R, Diallo S, Sakho A. Infanticide in Senegal: results from an exploratory mixed-methods study. Sex Reprod Health Matters. 2019;27(1):1624116. 10.1080/26410397.2019.1624116 31533577 PMC7888053

[30] Glynn AN. What Can We Learn with Statistical Truth Serum?Design and Analysis of the List Experiment. Public Opin Q. 2013;77(S1):159–72. 10.1093/poq/nfs070

[31] Blair G, Imai K. Statistical Analysis of List Experiments. Political Analysis. 2012;20(1):47–77.

[32] Blair G, Imai K. list: Statistical Methods for the Item Count Technique and List Experiment. The Comprehensive R Archive Network (CRAN); 2010.

[33] UNICEF. Progress for every child in the SDG era. New York, NY: United Nations Children’s Fund, Data and Analytics Section DoD, Research and Policy; 2018 March 2018.

[34] Menstrual regulation and unsafe abortion in Bangladesh. New York, NY: Guttmacher Institute; 2017. 23155545

[35] Levandowski BA, Kalilani-Phiri L, Kachale F, Awah P, Kangaude G, Mhango C. Investigating social consequences of unwanted pregnancy and unsafe abortion in Malawi: the role of stigma. Int J Gynaecol Obstet. 2012;118 Suppl 2:S167–71. 10.1016/S0020-7292(12)60017-4 22920622

[36] Archer N, Finden A, Pearson H. The laws, trials and imprisonment for abortion in Senegal. International Campaign for Women’s Right to Safe Abortion; 2018.

[37] Blair G, Coppock A, Moor M. When to Worry about Sensitivity Bias: A Social Reference Theory and Evidence from 30 Years of List Experiments. American Political Science Review. 2020:1–19. 10.1017/s0003055420000374

[38] Seck M. Au Sénégal, les cas d’infanticides posent la question de la légalisation de l’avortement. Le Monde Afrique. 2017 September 17, 2017. Available from: https://www.lemonde.fr/afrique/article/2017/09/17/au-senegal-les-cas-d-infanticides-posent-la-question-de-la-legalisation-de-l-avortement_5186962_3212.html.

[39] Geubbels E. Epidemiology of Maternal Mortality in Malawi. Malawi Medical Journal: The Journal of Medical Association of Malawi. 2006;18(4):206–25. 10.4314/mmj.v18i4.10923 27529012 PMC3345624

[40] Jackson E, Johnson BR, Gebreselassie H, Kangaude GD, Mhango C. A strategic assessment of unsafe abortion in Malawi. Reprod Health Matters. 2011;19(37):133–43. 10.1016/S0968-8080(11)37563-5 21555094

[41] Munthali A, Chimbiri A, Zulu E. Adolescent Sexual and Reproductive Health in Malawi: A Synthesis of Research Evidence. New York: The Alan Guttmacher Institute; 2004. Contract No.: 15.

