# Supplementary material for: Reducing underreporting of abortion in surveys: Results from two test applications of the list experiment method in Malawi and Senegal
Source: PLoS One. 2021 Mar 3;16(3):e0247201. doi: 10.1371/journal.pone.0247201 (PMC7928519; doi:10.1371/journal.pone.0247201)
Supplement: S1 Table — The objective of this analysis is to identify evidence for a design effect in either list. (DOCX) [file pone.0247201.s001.docx]

**S1 Table. Detailed assessment of weighted response proportions by number of reported items to list experiment questions, by list, among respondents in Malawi (n = 810).** The objective of this analysis is to identify evidence for a design effect in either list.

| **List A** |  |  |  |  |  |  |  |
| --- | --- | --- | --- | --- | --- | --- | --- |
|  |  | **Number of Reported Items** | | | | |  |
| **Estimated proportion** | **Source** | **0** | **1** | **2** | **3** | **4** | **Sum** |
| Row 1 | List with abortion | 0.022 | 0.341 | 0.502 | 0.127 | 0.009 | 1.000 |
| Row 2 | Proportion at least* | 1.000 | 0.978 | 0.637 | 0.135 | 0.008 | - |
| Row 3 | List without abortion | 0.021 | 0.356 | 0.464 | 0.159 | 0.000 | 1.000 |
| Row 4 | Proportion at least* | 1.000 | 0.980 | 0.623 | 0.159 | 0.000 | - |
| Row 5 | Row 2 minus Row 4 | 0.000 | -0.002 | 0.014 | -0.024 | 0.008 | -0.004 |
|  |  |  |  |  |  |  |  |
| **List B** |  |  |  |  |  |  |  |
|  |  | **Number of Reported Items** | | | | |  |
| **Estimated proportion** | **Source** | **0** | **1** | **2** | **3** | **4** | **Sum** |
| Row 1 | List with abortion | 0.013 | 0.453 | 0.441 | 0.087 | 0.006 | 1.000 |
| Row 2 | Proportion at least* | 1.000 | 0.988 | 0.535 | 0.094 | 0.006 |  |
| Row 3 | List without abortion | 0.007 | 0.448 | 0.482 | 0.063 | 0.000 | 1.000 |
| Row 4 | Proportion at least* | 1.000 | 0.993 | 0.545 | 0.063 | 0.000 |  |
| Row 5 | Row 2 minus Row 4 | 0.000 | -0.006 | -0.011 | 0.030 | 0.006 | 0.020 |
