# Supplementary material for: Reducing underreporting of abortion in surveys: Results from two test applications of the list experiment method in Malawi and Senegal
Source: PLoS One. 2021 Mar 3;16(3):e0247201. doi: 10.1371/journal.pone.0247201 (PMC7928519; doi:10.1371/journal.pone.0247201)
Supplement: S2 Table — The objective of this analysis is to identify evidence for a design effect in either list. (DOCX) [file pone.0247201.s002.docx]

**S2 Table. Detailed assessment of weighted response proportions by number of reported items to list experiment questions, by list, among respondents in Senegal (n = 1016).** The objective of this analysis is to identify evidence for a design effect in either list.

| **List A** |  |  |  |  |  |  |  |
| --- | --- | --- | --- | --- | --- | --- | --- |
|  |  | **Number of Reported Items** | | | | |  |
| **Estimated proportion** | **Source** | **0** | **1** | **2** | **3** | **4** | **Sum** |
| Row 1 | List with abortion | 0.048 | 0.358 | 0.410 | 0.154 | 0.030 | 1.000 |
| Row 2 | Proportion at least* | 1.000 | 0.952 | 0.594 | 0.184 | 0.030 | - |
| Row 3 | List without abortion | 0.047 | 0.378 | 0.391 | 0.184 | 0.000 | 1.000 |
| Row 4 | Proportion at least* | 1.000 | 0.953 | 0.575 | 0.184 | 0.000 | - |
| Row 5 | Row 2 minus Row 4 | 0.000 | -0.001 | 0.019 | 0.000 | 0.030 | 0.048 |
|  |  |  |  |  |  |  |  |
| **List B** |  |  |  |  |  |  |  |
|  |  | **Number of Reported Items** | | | | |  |
| **Estimated proportion** | **Source** | **0** | **1** | **2** | **3** | **4** | **Sum** |
| Row 1 | List with abortion | 0.063 | 0.289 | 0.448 | 0.184 | 0.016 | 1.000 |
| Row 2 | Proportion at least* | 1.000 | 0.937 | 0.648 | 0.200 | 0.016 |  |
| Row 3 | List without abortion | 0.053 | 0.266 | 0.532 | 0.135 | 0.014 | 1.000 |
| Row 4 | Proportion at least* | 1.000 | 0.947 | 0.681 | 0.149 | 0.014 |  |
| Row 5 | Row 2 minus Row 4 | 0.000 | -0.010 | 0.033 | 0.051 | 0.002 | 0.010 |
